# The Christian Science Bubble

**Published:** 1907-12

**Authors:** 


### EDITORIAL NOTES AND COMMENTS.

The Business Office of the JOURNAL-RECORD is 1 1-2 to 5 1-2 South Broad Street.

The Editorial Office is 318-319-320 The Prudential.

Address all Business Commucications to Dr. George Brown, Manager.

Make remittances payable to THE JOURNAL-RECORD OF MEDICINE.

On matters pertaining to the Editorial and Original communications, address Dr. Bernard Wolff, Atlanta.

Reprints of original articles will be furnished at cost price. Requests for the same should always be made on THE MANUSCRIPT.

We will present, postpaid, on request, to each contributor of an original article, twenty (20) marked copies of THE JOURNAL-RECORD OF MEDICINE containing such article

### THE CHRISTIAN SCIENCE BUBBLE.

The Magazines have devoted much space during the past few months to the old flimsy fake, —"Christian Science." Various articles, chiefly by women, have appeared, some coldly critical, some warmly laudatory, some temperate in statement and judgment.

In a recent number of one of the best of the cheap magazines, Mrs. Eddy, the head of the cult, perhaps to let the public know that she is still alive, and perhaps to summarize her creed, which has been so elaborately discussed, delivers herself of a string of moral platitudes worthy of a page from Clarissa Harlowe, or one of the instructive dialogues of Sanford and Merton. Mrs. Eddy shoggles along somewhat in this manner:— Man is material, God, *good*, is immaterial.— The immaterial dominating the material is alone admirable, alone leads to goodness, health, eternal life. Death is not a consummation, an abrupt change to perfection from a condition of imperfection. Preliminary training for death is necessary. The practice of benevolence, goodness, charity, truth in life and life in truth, with a little apple pie or a dish of cabbage thrown in now and then according to taste, or a drink of corn to drive out the cold, or of beer to dispel the warmth according to season; these things are they that conduce to goodness, happiness, health—are all steps in the pathway of life leading to death and immortality on the barred side.

It is strange that since Christ walked on earth in the flesh and taught at such a fearful sacrifice and with such a death of agony, the way of life and of eternity, people have gone into the dusk content with their belief that at this late day it should become necessary for this tremendous problem of existence or of non-existence to receive its exigesis at the hands of a flat-chested multi-married egoist, impregnated, so far as the narrow scope of her mind and opportunities permitted, with all the heresies of medicine and philosophy.

The cold narrative of her life shows a fixed determination toward self-advancement, regardless of truth, loyalty, gratitude, honesty or of any of the nobler attributes which should adorn anyone who claims to possess those qualities which Christ alone possessed. She put all things under her feet, bent every plastic nature to her own selfish ends. In return for hospitality, she attempted incendiarism; those that aided her most, she most traduced; those who were true to her, she scorned and contemned.—This is the sweet-minded, clear-conscienced moralist, who points out to thousands of fatuous disciples the way to health and happiness and heaven. For every stone cast at her, she turns her beatified gaze to high Heaven, with the uplifted look of a St. Stephen, and the hand that threw it becomes that of a blasphemer.

We may not see the end of this fallacy, but time will surely deal with it as it always has dealt with pretentious sham. It is a creed applicable to certain unstable temperaments, credulous of mystery, incredulous of things as they are—the temperament that feeds the clairvoyant, mind-reader, mental healer and such jugglers with the alleged occult and mystic.

The only quarrel which doctors have with this cult, besides an utter contempt for it and all its adherents, lies in its light denial of the existence of all that is demonstrably true, of established fact and verified phenomenon. The facts of nature are set aside with an easy indifference to proof in a manner exasperating enough to make an angel in heaven lean over the bulwarks and drop a chunk of masonry on the heads of these sacrosanct imbeciles.

The cult has spread like rot in a barrel of apples, until all with soft spots have been attacked. But it will be its own executioner, and will, by specious claim and unfounded theory, lose what little sanity it ever possessed and then follow the course of all defectives, whether man or philosophy,—Quis Deus vult perdere, prius dementat.

The world has a long time yet to roll before it burns to a cinder, and its rotary motion in times past has flicked into space much more adhesive rubbish than Christian Science.

